# Mindfulness and work addiction among young employees: the mediating roles of cognitive reappraisal and perfectionism

**DOI:** 10.3389/fpsyt.2025.1631792

**Published:** 2025-06-23

**Authors:** Gao Zheng, Jiafan Sheng, Huilin Wang, Ziqing Xu

**Affiliations:** ^1^ Institute of Science Innovation and Culture, Rajamangela University of Technology Krungthep, Bangkok, Thailand; ^2^ School of Business, Hunan University of Science and Technology, Xiangtan, China; ^3^ Business School, Guangdong Ocean University, Yangjiang, China

**Keywords:** mindfulness, cognitive reappraisal, perfectionism, work addiction, young employees

## Abstract

**Introduction:**

Work addiction has become a growing concern among young employees in China, fueled by an “always-on” work culture that has been amplified by the rise of digital technologies. This study investigates the psychological mechanisms that contribute to work addiction, focusing on mindfulness, cognitive reappraisal, and perfectionism. Understanding these mechanisms is crucial for developing effective strategies to combat work addiction, particularly in the context of the increasingly digital and competitive work environment in China.

**Methods:**

An online survey was administered to 362 young employees in southern China using convenience and snowball sampling methods. Data was analyzed using structural equation modeling (SEM) with the software SmartPLS 4 to examine the relationships between mindfulness, cognitive reappraisal, perfectionism, and work addiction. Mediation analysis was conducted to test the indirect effects of cognitive reappraisal and perfectionism on the relationship between mindfulness and work addiction.

**Results:**

The results revealed that mindfulness was positively associated with cognitive reappraisal and negatively correlated with perfectionism. Cognitive reappraisal was found to be negatively related to work addiction, while perfectionism showed a positive relationship with work addiction. Mediation analysis confirmed that both cognitive reappraisal and perfectionism mediated the relationship between mindfulness and work addiction.

**Discussion:**

This study highlights the role of mindfulness as a key factor in reducing work addiction among young employees. By enhancing cognitive reappraisal and reducing perfectionism, mindfulness can effectively mitigate the psychological drivers of work addiction. The findings suggest that organizations can implement mindfulness-based interventions to improve emotional regulation and work-life balance among young employees, ultimately helping to reduce work addiction and its associated negative impacts. The study also underscores the importance of conducting research in diverse cultural settings to further understand the global relevance of these psychological mechanisms.

## 1 Introduction

With increasing digitalization, young employees in China, especially in large cities, often prioritize career success, dedicating excessive time to work to stand out in a competitive job market (1). The widespread use of smartphones and social media has blurred the line between work and personal time, leading to an “always-on” work culture (2). This makes it difficult for them to relax, contributing to work addiction and overwork. Consequently, their social and recreational activities are limited, increasing psychological pressure and feelings of isolation (3). Long-term overwork can negatively affect mental health, causing anxiety, depression, and insomnia, while physical health issues like burnout and chronic diseases may also arise (4).

Work addiction, or workaholism, is a compulsive behavioral pattern where individuals excessively engage in work, detrimentally impacting health, social relationships, and personal well-being, often using work as a coping mechanism (5). It is characterized by excessive reliance on work, can have several negative consequences (6). Also, it is linked to mental health issues such as anxiety and depression, as individuals struggle to relax and may experience emotional breakdowns (7). Physically, overwork leads to burnout, insomnia, and other health problems, while also increasing the risk of chronic diseases (8). Work addiction also harms interpersonal relationships, causing social isolation and family conflicts (9). Although workaholics often believe they are productive, excessive work can reduce efficiency, impair decision-making, and lead to career burnout (5). Ultimately, work addiction lowers life quality by limiting time for self-care and personal interests. Addressing this issue through timely intervention is essential.

The Chinese government and companies have implemented measures to combat work addiction. The government has strengthened labor law enforcement, ensuring reasonable working hours and rest days, with increased supervision to prevent overwork (10). Companies are promoting work-life balance, especially in tech firms, where the “996 culture” has prompted flexible hours and remote work, “996 culture” refers to a work schedule commonly practiced in some Chinese companies, particularly in the tech industry, where employees are expected to work from 9 a.m. to 9 p.m., six days a week. This culture has been widely criticized for contributing to employee burnout and poor work-life balance (11). Growing attention to mental health has led companies to offer psychological support and stress management. Corporate cultures are shifting toward more humane management, reducing overtime (12). Additionally, some companies have established health-conscious workplace standards, such as rest areas and regular health checks, to mitigate work addiction (13).

Mindfulness, as an effective self-regulation tool, can alleviate and prevent work addiction from multiple perspectives (14). Firstly, mindfulness enhances self-awareness, allowing individuals to recognize their work-related behavior patterns and psychological states (15). When employees become aware of over-investing in work, mindfulness guides them to reflect and make timely adjustments, thus preventing unconscious overworking. Secondly, mindfulness helps improve work-life balance by helping employees distinguish between work and personal life boundaries, reducing work’s encroachment on personal time, and preventing health issues caused by overwork (16). Additionally, mindfulness helps alleviate perfectionism by promoting acceptance of imperfection and reducing excessive expectations, thereby mitigating work addiction driven by the pursuit of perfection (17). Finally, mindfulness enhances stress coping abilities by increasing awareness of the present moment, enabling individuals to better regulate their emotional responses under pressure, avoid emotional overload, and effectively recognize and address work-related stress, preventing prolonged high-pressure states (18).

The issue of work addiction among young employees has gained significant academic attention, focusing on its definition (19), psychological factors (20), consequences (21), and interventions (22). While research has explored work addiction’s impact on employees, there is limited research specifically on young employees, particularly regarding work stress, career, and societal expectations (23). Existing research mainly addresses short-term effects, with a lack of long-term studies. This study aims to examine the psychological mechanisms of work addiction in young employees, focusing on the role of cognitive reappraisal, perfectionism, and mindfulness interventions. The research questions include: (1) What are the key psychological factors (mindfulness, cognitive reappraisal, and perfectionism) associated with work addiction among young employees? (2) How do mindfulness, cognitive reappraisal, and perfectionism relate to each other in the context of work addiction? (3) What is the role of mindfulness in reducing work addiction among young employees?

## 2 Literature review and hypothesis development

### 2.1 Concept of variables

#### 2.1.1 Mindfulness

Mindfulness is a mental state characterized by the deliberate focus on the present moment with an attitude of acceptance, awareness, and non-judgment (24). It involves the ability to observe one’s thoughts, emotions, bodily sensations, and the surrounding environment as they occur, without attempting to change or escape them (25). This practice fosters a heightened awareness of both internal and external experiences, where individuals can observe their reactions without becoming overly involved in them or reacting impulsively (26). The fundamental concept of mindfulness is rooted in cultivating a balanced and non-reactive awareness of the present, which allows individuals to maintain clarity and emotional resilience, even when confronted with challenging situations (27). The origins of mindfulness can be traced to Buddhist traditions, where it is an integral part of meditation practices aimed at achieving enlightenment and reducing suffering. However, mindfulness has gained widespread recognition and application in modern psychological and therapeutic practices, particularly in the fields of stress reduction, emotional regulation, and mental health improvement. In recent decades, psychological models of mindfulness have evolved, with programs such as Mindfulness-Based Stress Reduction (MBSR), developed by Jon Kabat-Zinn, demonstrating its effectiveness in clinical and non-clinical settings alike (28). These programs emphasize cultivating mindfulness as a way to improve individuals’ ability to cope with stress, reduce symptoms of anxiety and depression, and enhance their overall emotional well-being.

Research has consistently shown that mindfulness can significantly enhance emotional regulation (29). Studies have found that mindfulness training is effective in reducing emotional reactivity and improving the ability to respond to stressful situations in a calm and measured manner (25). This is particularly beneficial in managing emotions like anxiety, depression, and anger, which can be exacerbated by an inability to regulate emotional responses. Mindfulness has also been shown to reduce stress levels, partly due to its focus on the present moment, which can prevent individuals from becoming overwhelmed by future concerns or past regrets (30). In addition to emotional regulation, mindfulness has been linked to improvements in mental health and overall well-being. It has been found to increase psychological flexibility, allowing individuals to accept and engage with their thoughts and feelings without becoming excessively attached or distressed by them (31). This, in turn, promotes a sense of well-being and can help individuals develop a more balanced and compassionate relationship with themselves (28). Mindfulness also enhances self-awareness, helping individuals gain insight into their own emotional patterns and behaviors, which can lead to healthier, more adaptive coping strategies (32).

#### 2.1.2 Cognitive reappraisal

Cognitive reappraisal is a central strategy within the broader field of cognitive emotion regulation, which refers to the ways in which individuals manage and modify their emotional responses to external stimuli or internal experiences (33). Cognitive reappraisal specifically involves changing the way one thinks about or interprets a potentially distressing situation in order to alter its emotional impact (34). This process is considered an adaptive emotional regulation strategy, as it enables individuals to reinterpret events or situations in a way that reduces their emotional intensity and helps them cope more effectively with stress or negative emotions (33). The concept of cognitive reappraisal is rooted in cognitive-behavioral theory, which posits that thoughts, emotions, and behaviors are interconnected (35). According to this framework, it is not the event itself that causes distress, but rather the way an individual perceives or appraises the event. Through cognitive reappraisal, individuals actively engage in changing their interpretations of situations, often reframing them in a more neutral, positive, or less threatening manner (36).

In addition to its emotional impact, cognitive reappraisal allows individuals to maintain psychological well-being by fostering a more flexible and adaptive response to stress (37). When individuals reframe their experiences, they are better equipped to view difficulties as manageable and less overwhelming. This shift in perspective can mitigate the intensity of negative emotions and prevent emotional overload (38). For example, cognitive reappraisal can help individuals reduce feelings of helplessness by viewing challenges as temporary and solvable rather than insurmountable. As a result, individuals are more likely to experience increased emotional resilience, greater psychological stability, and improved well-being (39). Research has shown that cognitive reappraisal is positively associated with improved emotional regulation. By reframing distressing situations, individuals can prevent emotional dysregulation, such as rumination, impulsivity, or emotional reactivity, which are common responses to stress and anxiety. This ability to regulate emotions more effectively is linked to better mental health outcomes, as individuals who engage in cognitive reappraisal tend to experience lower levels of depression, anxiety, and overall distress (40).

#### 2.1.3 Perfectionism

Perfectionism is a complex psychological trait that involves a pervasive tendency to set unrealistically high standards for oneself and others (41). Individuals who exhibit perfectionism are often driven by the desire to achieve flawlessness in every aspect of their lives (42). This drive for perfection can manifest in a range of behaviors, including an intense focus on details, an excessive need for control, and a constant striving for high performance across various domains, such as work, school, and personal life (41). Perfectionists frequently impose stringent demands on themselves, feeling that anything less than perfection is unacceptable. This perfectionistic mindset leads to significant self-criticism, as they may view minor mistakes or deviations from their high standards as failures. As a result, perfectionists often experience heightened anxiety, stress, and dissatisfaction with their achievements, which can have profound effects on their emotional well-being (43).

The concept of perfectionism can be broken down into two primary dimensions: self-oriented perfectionism and other-oriented perfectionism (44). Self-oriented perfectionism refers to the internal pressure individuals place on themselves to meet high standards. This dimension is often driven by an individual’s own expectations of what they should achieve and how they should perform. On the other hand, other-oriented perfectionism involves setting high standards for others and can lead to frustration or resentment when others do not meet these expectations. This form of perfectionism can cause interpersonal conflicts and negatively impact relationships, as perfectionists may be overly critical of others’ shortcomings. There is also a third dimension known as socially prescribed perfectionism, which is the belief that others, such as family members, friends, or society, hold unrealistic standards that one must meet. This form of perfectionism is often associated with feelings of pressure, inadequacy, and fear of judgment.

Research has consistently shown that perfectionism is strongly linked to negative psychological outcomes. One of the most significant consequences of perfectionism is its relationship with anxiety (45). Perfectionists often experience heightened levels of anxiety, particularly in situations where they perceive a potential for failure or judgment. This anxiety can become debilitating, leading to avoidance behaviors, procrastination, or a constant sense of unease. Moreover, the tendency to focus on flaws and imperfections makes perfectionists more vulnerable to depression, as they may feel hopeless or inadequate when they cannot achieve their idealized standards (46). Studies have shown that perfectionism is a risk factor for burnout, as individuals who are driven by perfectionistic tendencies are more likely to experience exhaustion and disengagement from tasks over time (47).

#### 2.1.4 Work addiction

Work addiction, also known as workaholism, is a behavioral pattern in which individuals compulsively engage in work to the extent that it interferes with other important aspects of life, such as health, social relationships, and personal well-being (48). At its core, work addiction is characterized by an inability to control one’s involvement in work-related activities, with work becoming a primary source of self-worth and a coping mechanism for managing stress or emotional difficulties (49). People who experience work addiction often prioritize their professional responsibilities over personal needs and relationships, which can lead to detrimental long-term consequences for their mental and physical health (50). Work addicts are driven by an overwhelming urge to work, often feeling anxious or restless when not working. This leads to behaviors such as extended work hours, persistent involvement in tasks even outside of work hours, and a tendency to overwork despite negative outcomes (51). Work addicts may feel guilty or uncomfortable when not working, and the idea of taking breaks or time off may seem foreign or unnecessary to them. They often exhibit work efficiency in the short term, but this is typically unsustainable, leading to burnout over time (52).

The concept of balance between work and life is another critical aspect of understanding work addiction. Healthy individuals are able to maintain a balance between their professional and personal lives, allocating time and energy to both career and personal interests (53). In contrast, work addicts tend to neglect aspects of their life outside of work. They often forget spending time with family and friends, engaging in hobbies, or focusing on their health in favor of work-related activities. This imbalance can lead to a breakdown in relationships, as loved ones may feel neglected or unimportant (54). Social isolation is a common consequence, as work addicts increasingly prioritize work over social interactions.

### 2.2 Hypothesis development

#### 2.2.1 Mindfulness, cognitive reappraisal, and perfectionism

Mindfulness and cognitive reappraisal are positively related, as both regulate emotions and cognition (55). Mindfulness enhances awareness and acceptance of the present moment, reducing emotional overreaction, while cognitive reappraisal changes the interpretation of situations to regulate emotions. Mindfulness helps individuals identify and adjust unfavorable emotional reactions, promoting the use of cognitive reappraisal (56). In contrast, mindfulness negatively relates to perfectionism, as perfectionists set high standards, leading to anxiety and self-criticism (57). Mindfulness encourages acceptance of imperfection and focus on the present, reducing the pressure and burden of perfectionism.

Finally, the relationship between cognitive reappraisal and perfectionism is also negative (58). While cognitive reappraisal can effectively reduce negative emotions, perfectionists tend to maintain high standards for themselves, making it difficult to fully accept their imperfections (59). This may result in reappraising situations as “not good enough” or “must be more perfect,” which exacerbates self-criticism and emotional distress, thus weakening the positive effect of cognitive reappraisal.

As a result, this study puts forward the following hypotheses:

Hypothesis 1 (H1): *There is a positive association between mindfulness and cognitive reappraisal.*

Hypothesis 2 (H2): *There is a negative association between mindfulness and perfectionism.*

Hypothesis 3 (H3): *There is a negative association between cognitive reappraisal and perfectionism.*

#### 2.2.2 Cognitive reappraisal, perfectionism, and work addiction

Cognitive reappraisal and work addiction are negatively related because cognitive reappraisal involves changing one’s interpretation of a situation to regulate emotions, helping individuals reframe stressful situations in a more adaptive manner (60). In the context of work addiction, individuals often perceive excessive work as necessary or unavoidable, and while cognitive reappraisal encourages flexible thinking, work addicts tend to have rigid work habits, making it difficult for them to effectively employ this strategy (61). As a result, cognitive reappraisal can counterbalance the emotional attachment to work addiction, leading to a negative relationship between the two.

On the other hand, perfectionism and work addiction are positively related. Perfectionists set excessively high standards for themselves and are highly motivated to meet these expectations (62). This drive for perfection often leads to excessive work involvement, as perfectionists feel compelled to overperform and control every aspect of their work. Therefore, perfectionism fosters the tendency to engage in work addiction, as perfectionists view work as a means to prove their worth and meet their high standards, which intensifies their work addiction (63).

As a result, this study puts forward the following hypotheses:

Hypothesis 4 (H4): *There is a negative association between cognitive reappraisal and work addiction.*

Hypothesis 5 (H5): *There is a positive association between perfectionism and work addiction.*

#### 2.2.3 Mediation effects

Cognitive reappraisal and perfectionism act as mediators in the relationship between mindfulness and work addiction. Mindfulness enhances awareness and acceptance of the present moment, reducing emotional overreaction and strengthening the ability to use cognitive reappraisal (64). This helps individuals reframe work-related stress, reducing emotional dependency on work and lowering the risk of work addiction. Additionally, mindfulness reduces perfectionism, which diminishes the drive for excessive work in perfectionists. Perfectionism often drives work addiction by encouraging overperformance to prove self-worth (65). Thus, mindfulness reduces both perfectionism and the drive for work addiction, explaining why higher mindfulness levels reduce work addiction.

As a result, this study puts forward the following hypothesis:

Hypothesis 6 (H6): *Cognitive reappraisal and perfectionism mediate the relationship between mindfulness and work addiction.*

**Figure 1:**
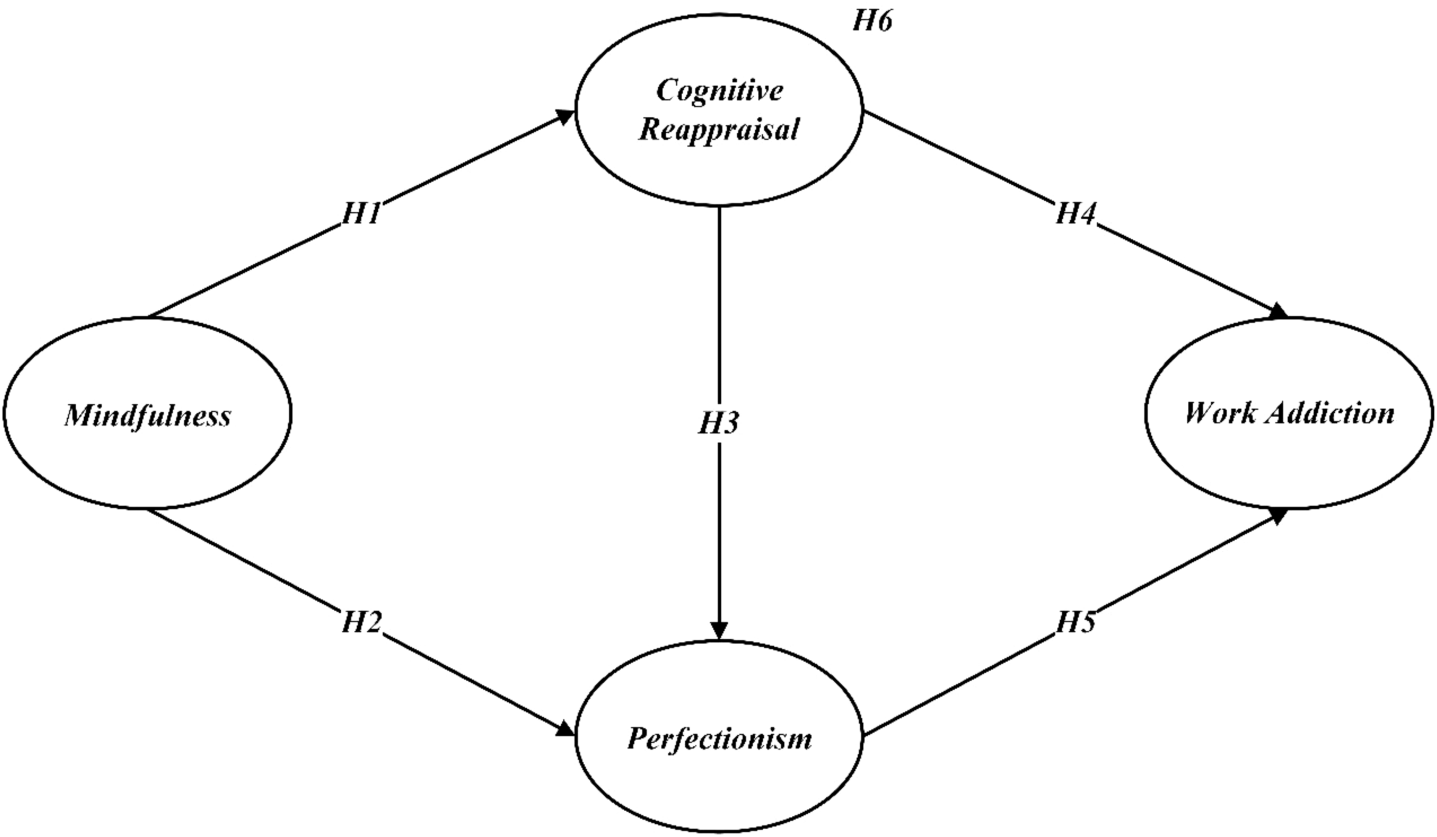
Hypothesized model.

The hypotheses are illustrated in **Figure 1**.

## 3 Methodology

### 3.1 Participants and procedures

This study employed an online questionnaire survey to gather data from young employees in the workplace. To ensure effective data collection, researchers utilized convenience and snowball sampling methods. With approval from relevant local authorities, they contacted young employees aged 18 to 35 in southern China. The recruitment process was facilitated through the social media platform WeChat, where researchers gauged participants’ interest and shared the questionnaire link via personal contacts, group chats, and Moments (WeChat’s public sharing feature), inviting them to participate. Prior to completing the survey, all respondents were informed of its purpose and provided their voluntary consent. Furthermore, following the snowball sampling approach, those who had completed the questionnaire were encouraged to invite their colleagues and friends to participate. The use of convenience and snowball sampling allowed for efficient access to a hard-to-reach population—young employees across various industries—within a limited time frame and under practical constraints. These methods facilitated data collection from diverse participants and leveraged existing social networks to increase sample size. The survey was hosted on Wenjuanxing (www.wjx.cn), a widely used online survey platform in China. A total of 568 questionnaires were distributed, yielding 362 valid responses, resulting in an effective response rate of 63.7%.

**Table 1** outlines the demographic characteristics of the 362 young employees included in this study: (1) The gender distribution was relatively balanced, with 45.3% male and 54.7% female respondents. (2) In terms of age, the majority fell within the 26–35 age group, accounting for 74.4%. (3) Regarding education, 96.4% of respondents held a bachelor’s degree or higher, indicating a highly educated sample. (4) Income levels were mainly concentrated in the 5,000-12,000 CNY range, with 33.1% earning 5,000-8,000 CNY and 35.4% earning 8,000-12,000 CNY. (5) In terms of sleep duration, 55.8% reported sleeping 5–7 hours per day. (6) 63.8% of respondents regularly engaged in leisure activities, indicating a relatively high participation rate.

**Table 1:** Demographics of sample.

Profiles		Frequency	Survey (%)
Gender	Male	164	45.3
	Female	198	54.7
Age	18-25	93	25.7
	26-35	269	74.3
Education level	College or below	13	3.6
	Bachelor’s degree	313	86.5
	Master’s degree or above	36	9.9
Monthly income level	Below 5000 CNY	80	22.1
	5000–8000 CNY	120	33.1
	8000–12000 CNY	128	35.4
	Above 12000 CNY	34	9.4
Average daily sleep duration	Less than 5 hours	37	10.2
	5–7 hours	202	55.8
	More than 7 hours	123	34.0
Do you frequently participate in leisure activities (e.g., sports, entertainment, etc.)?	Yes	231	63.8
	No	131	36.2

### 3.2 Instruments

The questionnaire consisted of five sections. The first section asked respondents to provide demographic information, including gender, age, highest level of education, monthly income level, average daily sleep duration, and whether they regularly participated in leisure activities (e.g., sports, entertainment).

The second section of the survey utilized the Mindfulness Scale (66), consisting of ten items to assess respondents’ mindfulness. Sample items include: “I am able to maintain clear awareness of what I am doing.” This scale is measured using a five-point Likert scale, with response options ranging from 1 (Strongly Disagree) to 5 (Strongly Agree).

The third section of the survey utilized the Cognitive Reappraisal Scale (67), consisting of six items to assess respondents’ use of cognitive reappraisal. Sample items include: “When I want to feel more positive emotion (such as joy or amusement), I change the way I’m thinking about the situation.” This scale is measured using a five-point Likert scale, with response options ranging from 1 (Strongly Disagree) to 5 (Strongly Agree).

The fourth section of the survey utilized the Perfectionism Scale (68), consisting of six items to assess respondents’ levels of perfectionism. Sample items include: “If I fail at work/school, I am a failure as a person.” This scale is measured using a five-point Likert scale, with response options ranging from 1 (Strongly Disagree) to 5 (Strongly Agree).

The fifth section utilized the Bergen Work Addiction Scale (BWAS) developed by Andreassen et al. (19), comprising seven items to collect data on respondents’ work addiction. Sample items included: “Worked so much that it has negatively influenced your health?” The scale was measured using a five-point Likert scale, with response options ranging from 1 (Never) to 5 (Always).

The original instruments (Mindfulness, Cognitive Reappraisal, Perfectionism, and the Bergen Work Addiction Scale) were developed in English, we conducted a rigorous translation process. First, all items were translated into Chinese by two bilingual researchers with expertise in psychology and survey design. Then, a separate bilingual expert, blinded to the original versions, performed a back-translation into English. Discrepancies between the original and back-translated versions were discussed and resolved through consensus among the research team to ensure semantic and conceptual equivalence. In addition, a pilot test with 30 participants was conducted to assess the clarity and cultural appropriateness of the translated items. Minor revisions were made based on feedback before finalizing the questionnaire.

### 3.3 Data analysis

In this study, structural equation modeling (SEM) was employed in conjunction with PLS-SEM to examine the proposed model. SEM is a widely recognized method for analyzing latent variables in measurement models and evaluating relationships among latent constructs in structural models (69). Following the two-step modeling approach outlined by Anderson and Gerbing (70), both the measurement and structural models were assessed using SEM. SmartPLS 4 was selected for SEM due to several methodological and practical considerations. Partial least squares SEM (PLS-SEM) is particularly suitable for exploratory research and theory development, especially when the research model is complex and the sample size is relatively moderate. Unlike covariance-based SEM (e.g., AMOS, LISREL), PLS-SEM places fewer restrictions on data distribution and is more robust when assumptions such as multivariate normality are not fully met. Given the predictive and exploratory nature of this study and the sample size (N = 568), SmartPLS 4 provided a flexible and appropriate tool for estimating path relationships, assessing measurement reliability and validity, and examining mediation effects. Initially, the reliability and validity of the measurement instruments were evaluated, with the lowest Cronbach’s alpha coefficient recorded at 0.932, confirming the instruments’ strong reliability and validity. Subsequently, the maximum likelihood estimation method was utilized to test the associations among mindfulness, cognitive reappraisal, perfectionism, and work addiction. Moreover, 5000 bootstrap samples were applied to investigate the indirect effects between high-intensity emotional intelligence and psychological detachment. Lastly, the model’s effectiveness was assessed by analyzing the fit indices and path coefficients of the hypothesized model.

## 4 Results

### 4.1 Measurement model

The reliability and validity assessment of latent variables incorporated confirmatory factor analysis (CFA) through SmartPLS 4. All variables demonstrated Cronbach’s α values surpassing 0.8 (refer to **Table 2**), affirming robust internal consistency within the model structure as guided by Fornell and Larcker (71). Additionally, the average variance extraction (AVE) for each variable exceeded 0.6 (as noted in **Table 2**), surpassing the minimal acceptable threshold of 0.5. Furthermore, the composite reliability (CR) of each latent variable surpassed 0.8, underscoring the model’s robust convergent validity. The resilience of convergent validity across proposed models was well-established. Factor loadings from principal component factor analysis ranged from 0.844 to 0.898 (refer to **Table 2**), reinforcing the measurement model’s robust construct validity.

**Table 2:** Reliability and validity analysis.

Items	Factor loadings	Cronbach’s α	CR	AVE
*Mindfulness (MI)*		0.932	0.948	0.785
MI1	0.892			
MI2	0.876			
MI3	0.882			
MI4	0.895			
MI5	0.886			
*Cognitive Reappraisal (CR)*		0.941	0.953	0.772
CR1	0.881			
CR2	0.885			
CR3	0.888			
CR4	0.875			
CR5	0.868			
CR6	0.876			
*Perfectionism (PE)*		0.945	0.956	0.784
PE1	0.882			
PE2	0.898			
PE3	0.883			
PE4	0.897			
PE5	0.863			
*Work Addiction (WA)*		0.941	0.952	0.740
WA1	0.844			
WA2	0.861			
WA3	0.863			
WA4	0.871			
WA5	0.852			
WA6	0.874			
WA7	0.856			

Cα, Cronbach’s alpha; AVE, average variance extracted; CR, composite reliability.

According to Fornell and Larcker (71), diagonal values of the square root of AVE are bigger than inter-item correlation values, demonstrates discriminant validity’. Item loadings, alpha, AVE and CR values are shown in **Table 2**, while discriminant validity scores are shown in **Table 3**. The outcomes revealed that the model is appropriate for the structural model evaluation employed in the final analysis.

**Table 3:** Discriminant validity.

Constructs	CR	MI	PE	WA
CR	0.879			
MI	0.585	0.886		
PE	-0.317	-0.316	0.885	
WA	-0.340	-0.368	0.678	0.860

MI, Mindfulness; CR, Cognitive Reappraisal; PE, Perfectionism; WA, Work Addiction.

### 4.2 Structural model

The study evaluated the model fit using the standardized root mean square residual (SRMR). The SRMR value is a standardized residuals index determined by comparing the observed covariance with the hypothesized matrix. A range of SRMR values of 0.1 or lower is considered acceptable (72). Based on the results, the estimated SRMR value was 0.041, which is considered a satisfactory model fit.

**Figure 2:**
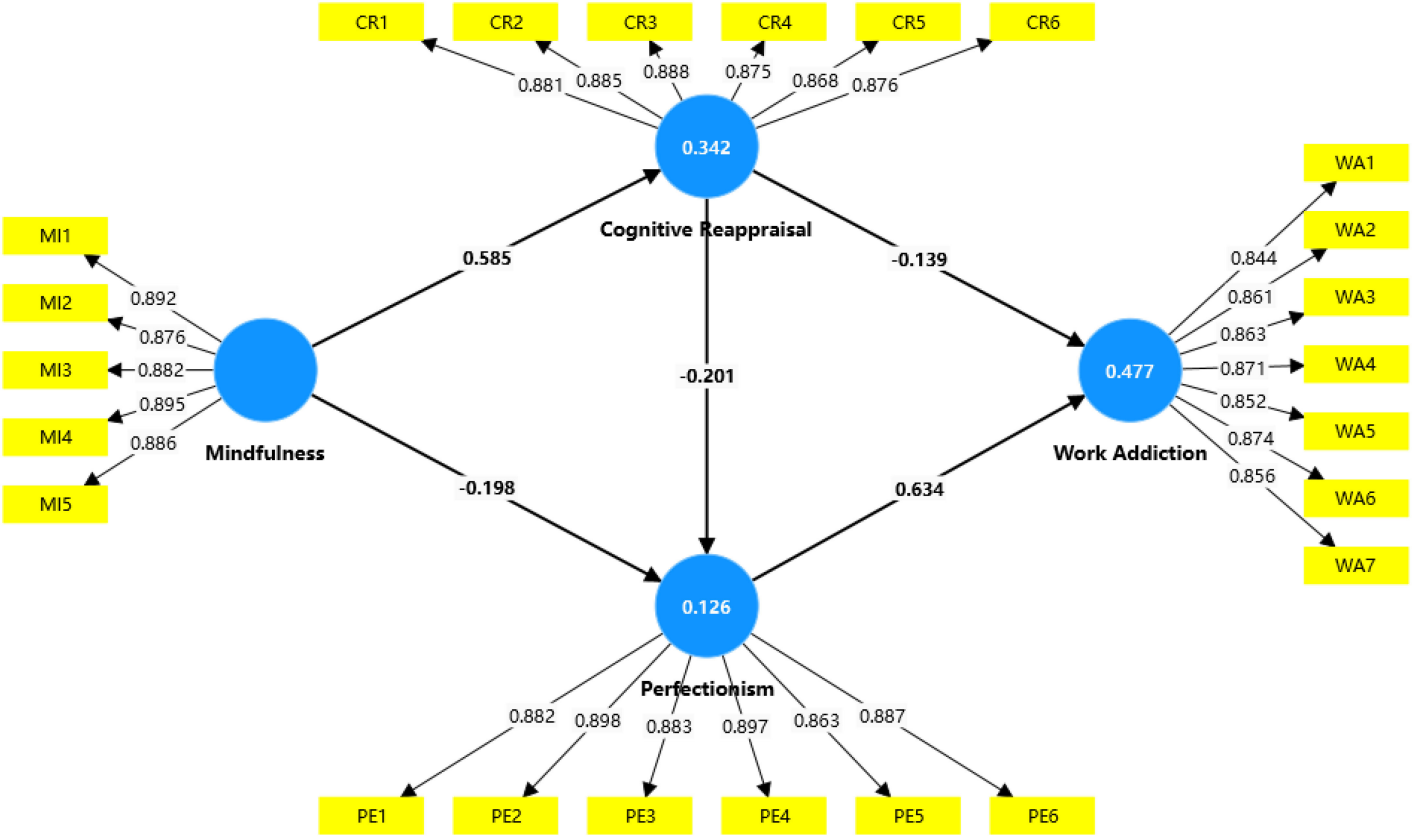
Structural path analysis model.

The structural path model in **Table 4**; **Figure 2** shows that the significant positive correlation between mindfulness and cognitive reappraisal (β = 0.585, t = 12.683, p < 0.001), supporting H1; and there was a significant negative correlation between mindfulness and perfectionism was (β = -0.198, t = 3.024, p < 0.001), supporting H2; and there was a significant negative correlation between cognitive reappraisal and perfectionism (β = -0.201, t = 2.919, p < 0.001), supporting H3; and there was a significant negative correlation between cognitive reappraisal and work addiction (β = -0.139, t = 3.254, p < 0.001), supporting H4; and the relationship between perfectionism and work addiction was statistically significant (β = 0.634, t = 13.922, p < 0.001), supporting H5.

**Table 4:** Path coefficients.

No.	Path	β>	Mean	T statistics	P values	LLIC	ULIC
H1	MI -> CR	0.585	0.586	12.683	0.000	0.485	0.667
H2	MI -> PE	-0.198	-0.197	3.024	0.000	-0.326	-0.068
H3	CR -> PE	-0.201	-0.202	2.919	0.000	-0.333	-0.062
H4	CR -> WA	-0.139	-0.139	3.254	0.000	-0.224	-0.058
H5	PE -> WA	0.634	0.636	13.922	0.000	0.534	0.715

MI, Mindfulness; CR, Cognitive Reappraisal; PE, Perfectionism; WA, Work Addiction.

The model’s explanatory power was evaluated by assessing the goodness of fit, determined through the strength of each structural path indicated by the R^2^ value for the dependent variable (73). According to Falk and Miller (74), an R^2^ value equal to or greater than 0.1 signifies a good fit. As shown in **Table 5**, all R^2^ values exceeded 0.1, confirming the model’s predictive capability. Additionally, Q^2^ was used to ascertain the predictive relevance of the endogenous constructs; a Q^2^ value above zero indicates predictive relevance. The results in **Table 5** demonstrate significant predictive power for the constructs.

**Table 5:** Outcomes of R^2^ and Q^2^ Values.

Constructs	R^2^	Adjusted R^2^	Q²
CR	0.342	0.340	0.260
PE	0.126	0.121	0.097
WA	0.477	0.474	0.350

CR, Cognitive Reappraisal; PE, Perfectionism; WA, Work Addiction.

The researchers proposed that work addiction impacts interpersonal conflicts through two mediating factors: emotional intelligence and psychological detachment. To test these mediation effects, bootstrapping methods were employed. As presented in **Table 6**, both cognitive reappraisal and perfectionism had a negative effect on the relationship between mindfulness and work addiction (standard indirect effect = -0.281, p < 0.001), thereby supporting H6.

**Table 6:** Mediation analysis.

No	Path	Original sample (O)	Sample mean (M)	Standard deviation (STDEV)	T statistics (|O/STDEV|)	P values
H6	Total indirect effect of MI ->WA	-0.281	-0.283	0.043	6.503	0.000
	Total Effect of MI ->WA	-0.281	-0.283	0.043	6.503	0.000
H6	Total indirect effect of WA -> IC	0.352	0.356	0.045	7.84	0.000
	Total Effect of WA -> IC	0.352	0.356	0.045	7.84	0.000

MI, Mindfulness; WA, Work Addiction.

WA, Work Addiction; IC, Interpersonal Conflicts.

### 4.3 Mediation analysis

The researchers hypothesized that work addiction influences interpersonal conflicts through two mediators: emotional intelligence and psychological detachment. This study tested the mediation effects using bootstrapping methods (Bollen & Stine, 1990). **Table 6** shows the emotional intelligence and psychological detachment significantly influenced the relationship between work addiction and interpersonal conflicts (standard indirect effect = 0.352, p < 0.001), supporting H6. The results suggest that respondents with lower work addiction who reported higher levels of emotional intelligence and psychological detachment engaged in less interpersonal conflicts.

## 5 Discussion

### 5.1 Theoretical contributions

This study provides a new perspective by exploring the relationships between mindfulness (55), cognitive reappraisal (61)., and perfectionism (63) in work addiction. Existing research often emphasizes the impact of emotional regulation strategies on work addiction, such as emotional intelligence and emotional regulation skills (28), and how perfectionism drives work addiction (47). However, less attention has been given to how mindfulness and cognitive reappraisal jointly reduce the risk of work addiction. The contribution of this study lies in introducing mindfulness and cognitive reappraisal to explore how they mediate the effect of perfectionism on work addiction. The mediation findings suggest that mindfulness may reduce perfectionistic tendencies by promoting non-judgmental awareness and self-acceptance. This helps individuals break away from rigid self-standards and excessive self-criticism. Theoretically, mindfulness supports emotional regulation and reduces cognitive fixation, which are central to perfectionism. Previous studies have not explicitly revealed the negative impact of mindfulness on perfectionism (21) or how cognitive reappraisal helps regulate work addiction, particularly in terms of how individuals reframe work situations (20). This study presents mindfulness as a crucial pathway for emotional regulation. The differences in findings may stem from variations in research methods, sample populations, and cultural backgrounds. While our findings are situated within the Chinese cultural context, they align with studies conducted in Western settings, where mindfulness has also been shown to reduce perfectionism and enhance psychological resilience. This suggests that the beneficial effects of mindfulness may hold across cultures, though the underlying mechanisms—such as the role of social expectations—may vary. Future cross-cultural research could further explore how cultural norms shape the pathways through which mindfulness influences perfectionistic tendencies.

This study lies in revealing how mindfulness and cognitive reappraisal mediate the relationship between perfectionism and work addiction, expanding the research scope on work addiction. Its replicability lies in providing a theoretical framework that can be applied to studies in other cultural contexts and emphasizing the potential of psychological regulation strategies in preventing work addiction, offering significant practical implications.

### 5.2 Practical implications

This study examines the role of mindfulness, cognitive reappraisal, and perfectionism in work addiction, with a particular focus on the psychological mechanisms underlying young employees’ experiences. The findings indicate that mindfulness significantly reduces the risk of work addiction by mitigating perfectionism and enhancing cognitive reappraisal. As digital transformation accelerates, young employees are increasingly influenced by an “always-on” work culture, making them more susceptible to excessive work engagement. The theoretical framework provided in this study suggests that organizations can implement mindfulness training to improve employees’ emotional regulation, enabling them to better manage work-life boundaries and effectively mitigate the negative consequences of work addiction.

Mindfulness training not only fosters emotional regulation but also strengthens employees’ psychological resilience and ability to cope with stress. The findings demonstrate that by cultivating greater awareness and acceptance of the present moment, employees can better manage workplace stressors and challenges, thereby reducing the likelihood of mental health issues such as anxiety and burnout resulting from excessive work involvement. Organizations can leverage these insights by incorporating regular mindfulness training programs to enhance employees’ self-awareness, ultimately improving both individual and team performance while lowering the incidence of work addiction.

This study underscores the critical role of organizational culture and management practices in human resource management. As work addiction becomes an increasingly pressing issue, more organizations are recognizing the importance of employee mental well-being and adopting strategies to promote work-life balance. The findings suggest that mindfulness not only alleviates work addiction but also helps employees reframe their perfectionistic tendencies, allowing them to adopt a healthier mindset when dealing with work-related stress. Based on these insights, organizations can implement more human-centered management practices, such as flexible work arrangements and remote work policies, to reduce employees’ work burden and enhance overall well-being.

This study underscores the critical role of organizational culture and management practices in human resource management. As work addiction becomes an increasingly pressing issue, more organizations are recognizing the importance of employee mental well-being and adopting strategies to promote work-life balance. The findings suggest that mindfulness not only alleviates work addiction but also helps employees reframe their perfectionistic tendencies, allowing them to adopt a healthier mindset when dealing with work-related stress. Based on these insights, organizations can implement more human-centered management practices, such as flexible work arrangements and remote work policies, to reduce employees’ work burden and enhance overall well-being.

### 5.3 Limitations

Despite providing valuable theoretical contributions, this study has several limitations. First, the sample is concentrated on young employees from southern China, which may not fully represent the situations in other regions or cultural contexts. While these approaches enabled efficient access to a relatively diverse sample of young employees and were practical under time and resource constraints, they may limit the representativeness of the population studied. Participants recruited through social media and personal networks may share similar demographic or psychological characteristics, potentially introducing selection bias. Future research could employ probability-based sampling techniques, such as stratified or cluster sampling, and expand data collection across different regions, industries, and employment settings.

Second, the study uses cross-sectional data, which limits the ability to examine long-term causal relationships between variables. Moreover, given the cross-sectional nature of this study, future longitudinal or experimental research is needed to confirm the effectiveness of mindfulness-based interventions and clarify causal relationships between key variables.

Third, although this study focused on individual-level psychological factors such as mindfulness and cognitive reappraisal, we acknowledge that other variables—particularly social support and work pressure—may also influence work addiction. Future studies could expand the model by incorporating these contextual and interpersonal factors to provide a more holistic understanding of work addiction.

Fourth, while various psychological measurement tools were employed, the reliance on self-reported data may introduce biases, such as social desirability or recall bias, potentially affecting the accuracy of the results. To minimize these effects, we ensured participant anonymity and used well-validated, neutrally worded scales. However, we acknowledge this limitation and suggest future studies incorporate additional data sources to enhance result validity.

Lastly, future research could benefit from a more diverse sample and longitudinal designs to better understand the enduring effects of mindfulness and cognitive reappraisal in different cultural settings. This would also help clarify whether the observed relationships hold across non-Chinese populations and under different cultural norms.

## 6 Conclusion

This study explores the relationships between mindfulness, cognitive reappraisal, perfectionism, and work addiction, providing insights into the psychological factors that may influence work addiction. The findings suggest that mindfulness is associated with lower levels of work addiction through its relationship with reduced perfectionism and enhanced cognitive reappraisal. To offer more specific guidance, organizations could implement brief daily mindfulness sessions, provide structured programmed like MBSR, involve leaders to model mindful behavior, and tailor content to address workplace stress or perfectionism. These steps can enhance the effectiveness and relevance of mindfulness interventions. However, given the cross-sectional design, the study does not establish causal relationships. Future research should investigate the longitudinal effects of these psychological mechanisms and explore their applicability in different cultural contexts.

## Data Availability

The raw data supporting the conclusions of this article will be made available by the authors, without undue reservation.
